# Supplementary material for: Glutamate Uptake Is Not Impaired by Hypoxia in a Culture Model of Human Fetal Neural Stem Cell-Derived Astrocytes
Source: Genes (Basel). 2022 Mar 12;13(3):506. doi: 10.3390/genes13030506 (PMC8953426; doi:10.3390/genes13030506)
Supplement: Supplementary file 1 [file genes-13-00506-s001.zip › genes-1598699-supplementary.pdf]

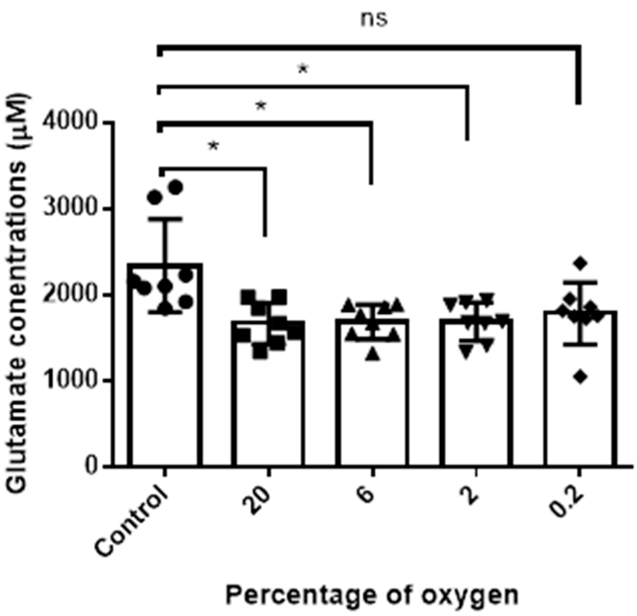

Figure S1. Glutamate concentration in supernatant (after 30 mins incubation) in astrocytes exposed to different concentrations of oxygen. Control is 2mM glutamate in HBSS. Data is visualized as Mean ± SD. * p-value <0.05; ns : not significant
